# Spatial mapping of the biologic effectiveness of scanned particle beams: towards biologically optimized particle therapy

**DOI:** 10.1038/srep09850

**Published:** 2015-05-18

**Authors:** Fada Guan, Lawrence Bronk, Uwe Titt, Steven H. Lin, Dragan Mirkovic, Matthew D. Kerr, X. Ronald Zhu, Jeffrey Dinh, Mary Sobieski, Clifford Stephan, Christopher R. Peeler, Reza Taleei, Radhe Mohan, David R. Grosshans

**Affiliations:** 1 Department of Radiation Physics, The University of Texas MD Anderson Cancer Center, Houston, Texas, U.S.A; 2 Department of Experimental Radiation Oncology, The University of Texas MD Anderson Cancer Center, Houston, Texas, U.S.A; 3 Department of Radiation Oncology, The University of Texas MD Anderson Cancer Center, Houston, Texas, U.S.A; 4 Center for Translational Cancer Research, Texas A&M Health Science Center, Institute of Biosciences and Technology, Houston, Texas, U.S.A

## Abstract

The physical properties of particles used in radiation therapy, such as protons, have been well characterized, and their dose distributions are superior to photon-based treatments. However, proton therapy may also have inherent biologic advantages that have not been capitalized on. Unlike photon beams, the linear energy transfer (LET) and hence biologic effectiveness of particle beams varies along the beam path. Selective placement of areas of high effectiveness could enhance tumor cell kill and simultaneously spare normal tissues. However, previous methods for mapping spatial variations in biologic effectiveness are time-consuming and often yield inconsistent results with large uncertainties. Thus the data needed to accurately model relative biological effectiveness to guide novel treatment planning approaches are limited. We used Monte Carlo modeling and high-content automated clonogenic survival assays to spatially map the biologic effectiveness of scanned proton beams with high accuracy and throughput while minimizing biological uncertainties. We found that the relationship between cell kill, dose, and LET, is complex and non-unique. Measured biologic effects were substantially greater than in most previous reports, and non-linear surviving fraction response was observed even for the highest LET values. Extension of this approach could generate data needed to optimize proton therapy plans incorporating variable RBE.

Interest in particle therapy, particularly proton therapy, has been increasing. The number of treatment centers in the United States alone is expected to double over the next 5–10 years. Although initial clinical results are promising, the rapid expansion of particle therapy is controversial given its high cost and the need for randomized trials to assess the clinical benefits of proton therapy compared with standard photon (X-ray) based treatments^1^,^2^,^3^.

Currently the widespread interest in proton therapy is driven by the physical properties of particle therapy, which allow greater sparing of normal tissues from excess radiation. The relevant physical properties stem from the fact that protons and other charged particles continuously lose energy as they traverse through tissue, with the rate of energy loss increasing as the particles slow. This phenomenon results in a dose deposition profile in which doses are low at the entrance into tissue, higher near the end of the range, and drop to near zero abruptly thereafter. The highest point of the dose deposition curve is known as the Bragg peak. In principle, these physical dose-deposition characteristics of particle therapy offer significant potential to enhance the therapeutic ratio compared with conventional modes of radiation therapy.

Although the physical properties of particles such as protons are well understood, much remains to be learned of their unique biologic effects. A large amount of research has demonstrated that particles generally have higher relative biological effectiveness (RBE) than photons (which by definition have an RBE of 1 when produced by Cobalt-60) towards the end of their range. This increased RBE indicates that particles are more biologically effective at inducing cell death than are photons, which underscores their potential for treating radiation-resistant tumors^4^,^5^. Heavier particles, such as carbon ions, have a significantly higher RBE than photons, with typical values ranging between 2 and 4 depending on the location along the beam path^6^,^7^. Protons, being relatively light particles, have an RBE closer to that of photons. In the current clinical practice of proton therapy, the RBE is assumed to have a generic, spatially invariant, constant value of 1.1^8^.

This assumption has been justified based on numerous *in vitro* and *in vivo* experiments carried out under relatively limited conditions using older delivery techniques (e.g., high doses per fraction, passive scattering)^8^. Although numerous, existing experimental data tend to be inconsistent and involve large uncertainties, a factor that is often used to justify the continued use of averaged RBE value of 1.1^9^. Even with high uncertainties and inconsistencies, many experiments have shown that the RBE of proton beams can vary considerably along the beam path and as a function of dose, but these potential variations in RBE are not accounted for in clinical treatment planning systems^10^,^11^,^12^. Thus, the concept of a generic RBE value for proton therapy is increasingly coming under scrutiny. Deeper understanding of the unique biologic effects of protons, combined with advances in planning delivery techniques, could considerably expand the therapeutic index of proton therapy.

Further, as delivery modalities progress, the opportunity arises to capitalize on the RBE variability of both protons and other ions. Historically, most clinical particle treatments were delivered with passive scattering technology, where a thin beam is scattered laterally and modulated longitudinally to produce a volume of uniform dose, comprising superposition of multiple Bragg peaks into what is known as the spread-out Bragg peak (SOBP)^11^. This volume is shaped by introducing materials into the beam to conform it to the three-dimensional shape of the target. In this delivery method, the highest-intensity Bragg curves contribute most of the dose to the formation of the distal edges of beams, which commonly lie within normal tissues beyond the target tumor volume.

Newer techniques use magnetically scanned thin pristine beams (“beamlets”) of particles with sequences of energies to produce dose distribution patterns to plan and deliver the most advanced form of particle therapy, intensity-modulated particle therapy (IMPT). IMPT involves sophisticated optimization techniques to adjust intensities and energies of incident pristine beams to balance the need to deliver maximum doses to tumor targets while maximally sparing nearby normal tissues.

IMPT has the potential to exploit the higher RBE of protons or other particles around the Bragg peak by preferentially placing the most biologically effective portions of the beam inside the target volume and away from normal structures. This is in contrast to current delivery techniques, in which the treatment is optimized solely to create a uniform dose in the target volume without consideration of the varying biological effectiveness of the particles in the beam. This preferential placement approach could significantly increase the differential between doses to target vs. normal tissues. However, the incorporation of variable RBE into IMPT optimization requires that accurate models be developed for computing RBE as a function of dose per fraction, linear energy transfer (LET), and tissue type, which in turn would require large amounts of accurate data on RBE. Unfortunately, data for developing these models are limited and have been difficult to obtain because of numerous complicating factors such as adequate access to beam time, non-standardized irradiations, variations in experimental techniques and reporting as well as the large span of reported biologic responses^9^,^13^.

In a recent comprehensive review, Paganetti systematically analyzed hundreds of published clonogenic data points to determine a relevant proton RBE^9^. That review stated that although the use of an RBE of 1.1 is acceptable for large clinical SOBPs, the literature does support increased RBE values of 1.15 at the center, 1.35 at the distal edge, and 1.7 at the distal falloff of an SOBP. Equally as important, the review highlights the large spread of the existing clonogenic data and suggests a need for experimental protocol standardization in addition to more complete reporting of fit parameters and errors. Although clearer data reporting is relatively straightforward, the ideal experimental protocol will require optimization owing to the multitude of complicating factors in a clonogenic assay alone that could contribute to the observed data spread.

In this report, we describe our combined use of basic particle physics and minimization of biological uncertainty to design a high-throughput system for improving the accuracy of the acquired biologic data as a function of dose and LET. We report preliminary results generated with actively scanned monoenergetic proton beams as a demonstration of the potential of such an approach. We anticipate that this method will continue to evolve to address multiple experimental needs, not only for proton biology but also for similar experiments with other ion species.

## Results

### Monte Carlo–based design of a customized device to accurately and efficiently map biologic effects

**Figure 1:**
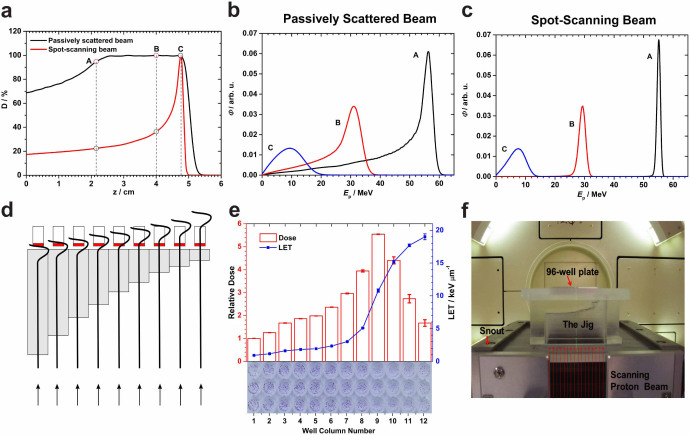
Rationale for using the scanned monoenergetic proton beams for biology experiments and design of the irradiation device. (a) Depth-dose profiles for a 79.7-MeV scanned proton beam vs. a matched passively scattered beam of the same range with a 3-cm spread-out Bragg peak (SOBP) in water. (b) Energy spectra of protons at three points A, B, C within the scattered beam marked in panel (a). (c) Corresponding energy spectra for the monoenergetic 79.7-MeV scanned beam. (d) Schematic diagram of the irradiation device (jig) concept illustrating the strategy for the column-by-column simultaneous irradiation of biological samples in the 96-well plate with protons at different points on the pristine Bragg curve. Gray bars indicate Lucite; red, culture medium. The stepped construction is designed to match the columns of a 96-well plate and serves to vary position along the Bragg curve, although only 9 columns are shown in the illustration and the step dimensions are not to scale. (e) Dose and LET distributions in the cell layers, positioned atop the jig, were computed using Monte Carlo simulations. The relative dose results shown were normalized to the entrance dose in column 1 in the 96-well plate. The LET shown is dose-averaged LET. The associated errors for both dose and LET were obtained from a sensitivity analysis of experimental setup uncertainties. The thickness of the 12 steps in the jig was selected according to the variations of dose and LET along the Bragg curve. Column 9 was aligned with the Bragg peak by inserting three films of thickness 268 µm each. An exposed and processed 96-well plate is shown at the bottom of the panel to illustrate the dose-LET effect of cell kill. (f) The jig directly mounted onto the scanning beam gantry. The 96-well plates are inserted into a precisely milled slot in the jig holder designed to minimize positioning errors. Protons are incident from below.

The spatial energy spectra, and hence the LET spectra, of a therapeutic proton beam depend on several factors including incident energy, SOBP width, and position of measurement within the SOBP as well as the machine-specific hardware used for scattering and range modulation. We compared the calculated energy spectra between passively scattered and scanned beams at three matched locations along the beam paths and found substantial differences between the two delivery methods (Fig. 1a–c). The broad energy spectra of passively scattered beams, particularly the long low-energy tails, could introduce significant uncertainty in the relationship between biological effect and LET.

To minimize the breadth of the energy and LET spectra, and to facilitate correlations of biologic effect with LET and dose, we developed a system using monoenergetic scanned proton beams. Because LET increases as a function of depth along the Bragg curve, first slowly and then rapidly, we used Monte Carlo (MC) simulations to design an apparatus (jig) to attenuate proton energy in a stepwise fashion from the incident energy to the end of the range (schematically illustrated in Fig. 1d). We chose 96-well plates to allow the simultaneous irradiation of biologic samples to multiple dose-LET combinations and automated plate processing. The irradiation jig was designed by grouping the 96 wells into 12 columns of 8 wells each, such that each of the columns are simultaneously exposed to a different combination of dose and LET, thus enabling the acquisition of 12 times the amount of data from a single exposure. All of the wells in a column are intended to receive the same dose-LET combination (Fig. 1e). The resulting design can be considered a multi-step range shifter consisting of 12 steps. The thickness of steps varies from 0 to a maximum value producing protons of increasing LET; the thickest steps irradiate the cells with the portion of the Bragg curve from just before to just beyond the Bragg peak. The jig was custom-fabricated from Lucite and directly mounts into the snout of the scanning beam gantry. The biologic sample plate is placed on top of the jig with the beam directed upwards (Fig. 1f). The thickness of the material interposed in the proton beam path initially varies in large steps and then in increasingly finer increments as the end of the proton range approaches and at points beyond. This approach was taken to increase the resolution in regions of high dose and LET gradients. MC calculations indicated that the jig did not appreciably alter the profiles of the energy spectra (Supplementary Fig. 1).

### Validation of spatial accuracy and irradiation of samples

**Figure 2:**
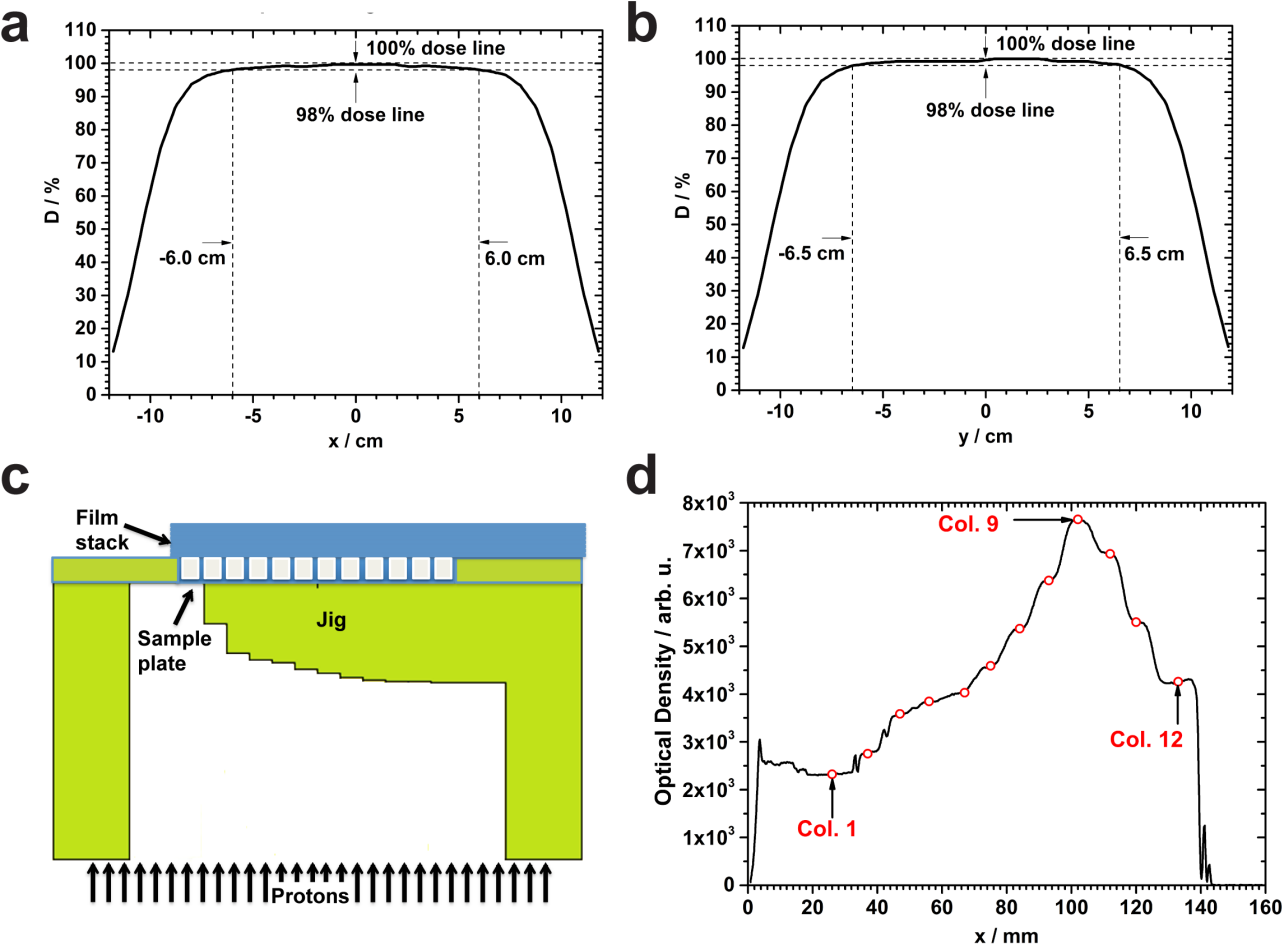
Uniformity of the scanning field and verification of distal edge placement. (a) Dose profile along the central longitudinal axis in the isocenter plane, resulting from a 20 × 20 cm^2^ uniform scan pattern with 3.5-cm full-width at half-maximum (FWHM) spots spaced 1 cm apart, measured with a 1,020 chamber MatriXX system (IBA I’mRT MatriXX, Schwarzenbruck, Germany). The field width between the 98% and 100% dose levels is 12 cm. (b) Dose profile along the central lateral axis in the isocenter plane. The field width between the 98% and 100% dose levels is 13 cm and is along the direction of the wells of columns; the uniformity over the 7.2-cm extent of the 8 wells in each column is 99%–100%. (c) Schematic of the experimental setup for range verification. A stack of 12 EBT3 films (each 268 µm thick) was placed directly on top of an empty 96-well plate and exposed. (d) Optical densitometry measurements of individual films made through the center of the well rows indicated that the Bragg peak was at well column 9 for this film, the fourth in the stack. In addition to verifying the accuracy of penetration of protons, this experiment allowed us to determine the number of films needed to position one of the columns (number 9) at the Bragg peak and have three points of measurement in the high gradient distal fall-off.

To verify the precision of the jig setup and radiation delivery in relation to the Bragg curve, a scan pattern consisting of 441 spots of a 79.7-MeV proton beam was generated to form a 20 × 20 cm^2^ field. The field was found to be uniform over an area of 12 × 13 cm^2^, large enough to cover the biological sample area of 10.8 × 7.2 cm^2 ^(Fig. 2a&b). The respective column doses to a simulated cell layer were also found to be within the statistical uncertainty of the MC simulations (±<1%). To determine the location of the Bragg peak, we exposed a stack of twelve EBT3 films (each 268 µm thick) placed on top of an empty 96-well plate inserted into the jig (Fig. 2c). Optical densitometry analysis revealed that the location of the Bragg peak position changed from film to film depending on the total intervening thickness. To allow three points of measurement in the high-dose gradient region beyond the Bragg peak, we sought to place the peak in the cell layer of column 9. We found that the peak was located at column 9 for the fourth film, indicating that insertion of three films below the sample plate would provide the desired shift (Fig. 2d). Subsequently, three EBT3 films were placed below the sample plate in all MC simulations and irradiations for clonogenic assays. In the presence of the jig, the dose varied across the columns of the plate from entrance to the peak by a factor of 5.5. Two subsequent validation tests were performed 2 months apart using a calibrated clinical plane-parallel ionization chamber and the jig setup. The ion chamber was inserted into a Lucite holder and irradiated atop the exact jig setup to be used for future biologic sample irradiation. The MC-predicted dose and ion chamber measurement were found to differ by 0.35%, which is within the expected setup uncertainty from our sensitivity analysis (Supplementary Fig. 2; Supplementary Table 2). This setup was then used to irradiate 96-well plates with biologic samples to a range of entrance doses, thus producing a set of samples exposed to a matrix of dose-LET combinations (Fig. 1e).

### Mapping variations in biologic effect with high-content automated assays

**Figure 3:**
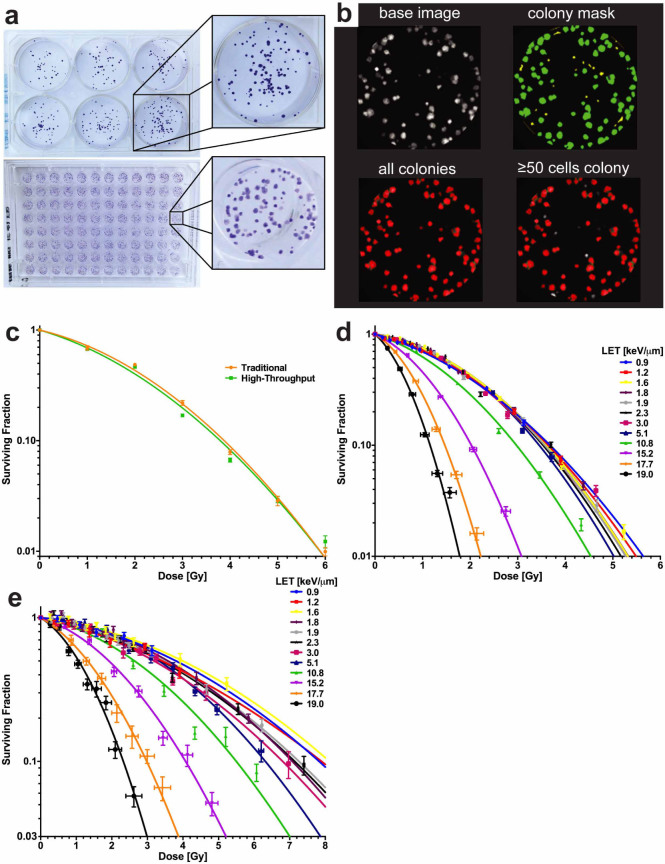
High-throughput clonogenic assays of H460 and H1437 NSCLC cells. (a) As a benchmark for our 96-well system, we compared cultures in that format, processed and counted with the IN Cell Analyzer 6000, with those in 6-well plates, counted manually. Characteristic images of each plate type are shown. (b) Representative images of a single well depicting high-content image processing. (c) Cell survival curves for individual plates of cells grown in 6-well or 96-well systems, exposed to ^137^Cs gamma irradiation, and scored by manual or automated processing. Curves were found to be not statistically different between the techniques (*P* = 0.315, extra sum-of-squares *F* test). Error bars represent standard error of the mean (s.e.m.). (d) Clonogenic survival plotted as a function of dose and LET for proton irradiation experiments with H460 cells. Error bars for dose were calculated by sensitivity analysis and SF with s.e.m. (e) Clonogenic survival as a function of dose and LET for proton experiments performed with H1437 cells. *P* < 0.0001 for comparisons of 0.9 keV/µm to ≥10.8 keV/µm, extra sum-of-squares *F* test.

To establish the feasibility of our approach and relate our findings to those of historical studies, we used dose-averaged LET-dependent cancer cell line clonogenic survival as the primary endpoint for our initial experiments. We adapted high-content screening techniques for higher throughput and used specific techniques to increase the accuracy of the data generated^14^. We first benchmarked the high-content system by comparing clonogenic assay results obtained from both the high-content and traditional method of manually counting colonies in 6-well plates of cultured H460 non-small cell lung cancer (NSCLC) cells (Fig. 3a&b). The H460 cell line was chosen for its exceptional clonogenicity and its doubling time, which we found to be 19 hours (in agreement with published values)^15^,^16^. Log-phase H460 cells were plated into either 96-well plates (0.33 cm^2^ growth area per well) or 6-well plates (9.5 cm^2^ growth area per well) from the same stock solution at a seeding density of 100 cells per well for the 96-well plates and a range of concentrations for the 6-well plates. The cells were allowed to attach and normalize for 8–10 hours before being irradiated. Radiation was delivered with a ^137^Cs irradiator. Three different passages of the cell line were used to calculate the average surviving fraction (SF) with a single 96-well plate at each dose level for the high-content method, or with 6 replicates for each dose per experiment for the traditional method. After irradiation, the cells were allowed to grow until sufficient colony formation was observed in the control conditions (5 days for the high-content system and 10–12 days for the traditional method) and then fixed and stained. Scoring, either by manual counting for the traditional method or with an IN Cell Analyzer 6000 for the high-content system (Fig. 3b), showed good correlation between techniques, with a non-statistically significant difference between the curves (*P* = 0.315, extra sum-of-squares *F* test; Fig. 3c).

We next processed plates for exposure to protons using the jig. Exponentially growing H460 cells were detached, automatically counted, diluted, and plated into 96-well plates. Plates were seeded at 100 cells per well from the same stock solution using an automated plater. After either photon (^137^Cs) or proton irradiation, plates were cultured until colony formation, at which time they were stained and prepared for readout and subsequent survival analysis. The results of our high-content proton experiment revealed that irradiation with increasing LETs resulted in a marked decrease in cell survival (Fig. 3d). This trend was either obscured or not present at the lower LET values in the plateau region of the Bragg curve, but it was especially pronounced for the LET values at and beyond the Bragg peak (Fig. 3d). Data fitting found that the SF at a dose of 2 Gy (SF2) for the H460 cells was 0.40 for the lowest LET tested (0.9 keV/µm) and 0.29 for protons with an LET of 10.8 keV/µm at the Bragg peak. Beyond the Bragg peak, in high-LET areas, we found still lower SF2 values of 0.10 for 15.2 keV/µm, 0.021 for 17.7 keV/µm, and 0.0037 for 19.0 keV/µm.

**Figure 4:**
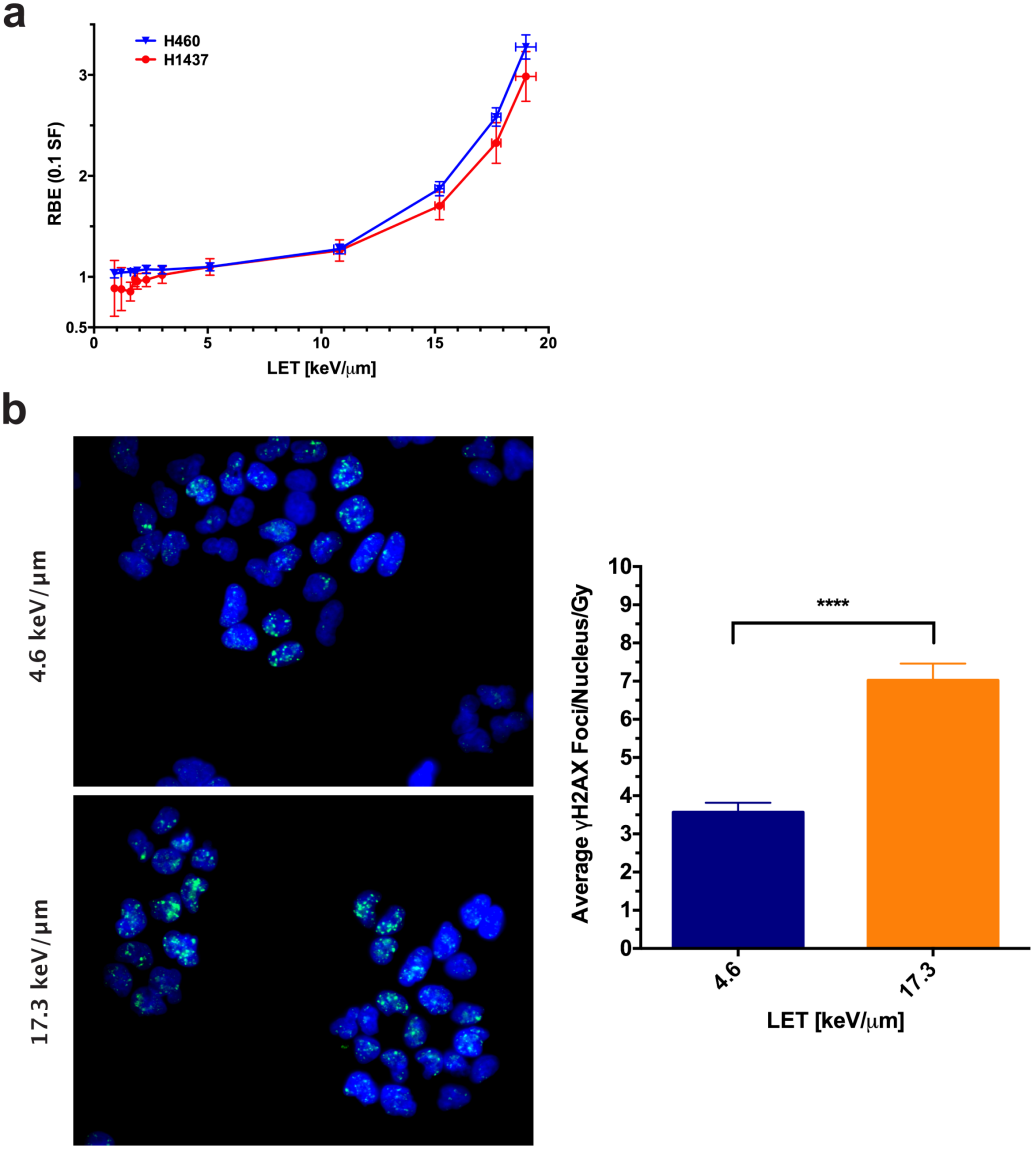
Preliminary analysis of biological assays. (a) RBE vs LET at 10% SF. A nonlinear trend between biological effect and LET was observed for both cell lines. RBE error was calculated by propagating the standard error of the α and β fits from Fig. 3 (Supplementary Table 1). (b) H460 cells were plated in glass-bottom 96-well plates, irradiated, and processed 2 hours later for γH2AX foci staining. Representative images are depicted. Comparing wells exposed to LETs and doses of 4.6 keV/μm, 2.9 Gy and 17.3 keV/μm, 1.7 Gy, the average nuclear foci per Gy was significantly increased in the high-LET samples (*P* < 0.0001, Mann-Whitney unpaired *t* test). Error bars represent the 95% confidence level.

These initial results with the H460 cell line prompted us to further assess our method. To obtain higher-dose data we used the p53-mutant NSCLC line, H1437, which is resistant to apoptosis^17^. A subsequent experiment with H1437 cells corroborated the trend seen with the H460 cells (Fig. 3e). Calculated SF2s were between 0.70 and 0.79 for the low LETs tested (0.9 to 5.1 keV/µm), 0.63 for 10.8 keV/µm, 0.47 for 15.2 keV/µm, 0.29 for 17.7 keV/µm, and 0.16 for 19.0 keV/µm using the data fits to the H1437 dataset. Table 1 contains detailed information on the α and β fit parameters and the recorded RBE values at 10% SF vs. ^137^Cs for the clonogenic survival plots. Plotting the RBE vs. LET for the two cell lines revealed a nonlinear response over the tested range where the RBE scaled in a biphasic manner (Fig. 4a).

To demonstrate the applicability of the developed system for use with assays other than clonogenic survival, we next compared DNA double-strand break induction (measured as γH2AX foci formation) between low- and high-LET regions in the H460 cells. As predicted, in high-LET regions, more foci were present 2 hours after irradiation (Fig. 4b). The overall dose-averaged number of γH2AX foci per nucleus was found to be significantly lower at 3.57 ± 0.25 for 4.6 keV/μm protons versus an average of 7.02 ± 0.44 foci per nucleus at 17.3 keV/μm (*P* < 0.0001, Mann-Whitney unpaired *t* test).

## Discussion

We found that an integrated physics and biology approach, coupled with high-throughput techniques, can be used to systematically map biologic responses to actively scanned proton beams as a function of dose and LET. In developing this method, we attempted to reduce sources of uncertainty while simultaneously increasing data output.

Clonogenic assays have been used to assess cellular reproductive integrity after an insult for more than 50 years^18^,^19^. Evolution of the exact method and understanding of the assay has produced a rich amount of relevant literature to draw upon. For each of our experiments, cultured cell concentrations were determined by using an automated cell counter, and all plates were seeded at a constant density from a single stock solution within 10–15 minutes of one another by using an automated plater. Use of a single seeding solution was intended to reduce potential errors in cell counting on colony formation. Even at the ideal theoretical limit of cell counting accuracy, the associated counting error for a standard cell solution is approximately 15%–30%^20^,^21^. This fact alone contributes intrinsic noise to clonogenic data and introduces compounding uncertainty when the number of cells per dose are counted separately.

All cell lines have inherent biological sensitivities to culturing conditions that may or may not affect the outcome of a clonogenic experiment. We attempted to remove or minimize any possible confounding factors from our colony readout as follows. Use of a single seeding solution per experiment requires a single multiplicity correction, as applicable^22^. Plating cells before irradiation and allowing them to reattach and recover from the stresses induced by plating kept our focus on the effect of radiation on the clonogenicity of exponentially growing cells^23^,^24^. Seeding cells after irradiation adds factors that contribute to the biological endpoint, resulting in increased uncertainty. Immediate post-irradiation plating in particular involves the effects of cell detachment during seeding while the cells are still repairing radiation-induced damage^25^. In addition to anchorage-dependent signaling and morphologic changes adherent cells undergo during detachment, enzymatic detachment solutions, such as trypsin, cleave membrane-bound adhesion molecules that function as major signalers in cellular stress responses, including apoptosis^26^,^27^,^28^,^29^. Post-irradiation cell detachment can further confound determination of RBE because photons and ions have different effects on cell adhesion and motility^30^,^31^,^32^. As such, immediate post-irradiation plating readouts involve coupling the effect of the radiation treatment with cellular reattachment, except in the plating efficiency control used to determine overall SF levels. Another complicating factor of post-irradiation plating is the possible contribution of potentially lethal damage repair to cell survival depending on the experimental conditions and timeline^19^,^33^,^34^,^35^. For this work, we used the minimum number of counting steps for a clonogenic assay, attempted to minimize relative errors by ensuring the exchangeability of inter-experiment plates, and removed complicating biological processes from the readout of colony formation.

Our high-throughput method presented here is not without its shortcomings. The initial jig design was intended to evenly sample the Bragg curve. However, because of the gradual increase in LET along the entrance plateau, followed by the rapid rise proximal to and just beyond the Bragg peak, the current jig design resulted in oversampling of low-LET points (< 5 keV/µm) and undersampling of LETs in the range of 5–10 keV/µm. Ideally, the design of the jig could be optimized for uniform LET sampling. The increased throughput gained by using the 96-well format comes at the cost of limiting the maximum number of cells that can be seeded without substantial colony overlap in high-SF plates to about 200^14^. The seeding density limitation restricts the range of measureable SFs, making achievable doses cell line-dependent. The infrastructure required for the high-throughput method is also greater than for the standard method and may not be universally available. The influence of the bystander effect on the clonogenic assay is unclear, so we cannot comment on any expected differential effects between the standard and high-throughput method^36^.

While no studies have been reported that demonstrate a method to map the biological effect of protons with this type of throughput, other groups have done similar rigorous investigations using traditional clonogenic assays to map combined LET-dose effects^11^,^12^,^13^,^37^. As previously noted, direct comparisons between studies are difficult because of differences between experiments; however, general observations can be made. Although recent studies have indicated that RBE and LET scale linearly, our results suggest that the relationship between LET and biologic effect may not be so straightforward^9^,^12^,^13^. In both cell lines tested, RBE increased rapidly with LET for values at and beyond the Bragg peak (>10 keV/µm), but this trend was reduced for the LETs measured from the entrance to the proximal portion of the Bragg peak (< 10 keV/µm). All SF data fits from our work to the linear-quadratic model produced curves with substantial ‘sub-lethal’ (β) components, resulting in nonlinear dose-responses even at the highest LETs (Table 1). Ubiquitous quadratic components are unusual, as an established hallmark of SF after high-LET irradiation includes α-dominated survival curves^6^,^9^,^12^,^37^,^38^. This observation results in a nonlinear response between RBE and LET (Fig. 4a). One possible explanation is that in many previous investigations, the LET values used to quantify the relationship between RBE and LET are determined by averaging over the broad spectrum of a passively scattered beam rather than the narrow LET spectrum of a scanned beam. Because different energy and LET spectra can coincidentally yield the same average value yet result in substantially different biologic effects, the dose-averaged LET used to estimate RBE may not correlate well with measured biologic results^39^. This averaging may obfuscate the LET effect of cell kill, especially at the end of range for charged particles, where the LET has a wide spread. For example, in the 12th column of the plate in the presented setup, MC simulation showed a proton LET range of 3 to 80 keV/µm, resulting in a dose-averaged LET value of about 19 keV/µm. Narrow LET spectra should help to further elucidate the relationship between RBE and LET.

The measured RBEs in the distal falloff region of the beam are considerably higher than those typically reported in the literature as well as the clinically used value of 1.1; however, such values are not unheard of or the highest reported^8^,^9^,^40^. Our results imply that the evaluation of biologic effect must ensure comprehensive characterization and suggest that additional studies using high-precision methods are required to develop accurate models of biologic effect.

Although applying the results of *in vitro* assays to *in vivo* models has its own complications, clonogenic survival is by far the most commonly used and well correlated cancer cell line assay for tumor control probability, with substantial evidence establishing the relation between the two methods^9^,^41^,^42^,^43^,^44^. The actual translation of the presented work towards preclinical evaluation of a biologically weighted treatment *in vivo* requires substantial work even in the simplest murine cancer model because of sensitivity to setup uncertainties and the small scale of the anatomy. Meanwhile, a much larger knowledge gap exists in the assessment of *in vivo* normal tissue radiation toxicity, where cellular clonogenicity is but one of many factors affecting organ response and function^45^,^46^,^47^. For complete biological optimization, normal tissue tolerances and responses must also be understood and quantified in the appropriate biological context for effective modeling.

Whereas clonogenic survival was the primary endpoint in the current study, this system can be easily modified to incorporate more advanced biologic methods and models. In particular, 3D tissue culture holds great promise to produce settings that better recapitulate an *in vivo* normal or tumor environment with corresponding tissue imitation^48^,^49^,^50^,^51^. Our approach may also serve as a platform for investigating other measures of biologic response as a function of LET, including DNA damage response, cell signaling, or epigenetic alterations. Moreover, the adaptation of other biologic models, facilitated by the high-content approach, will allow functional assays of surviving cells, which could be useful for investigating radiation-induced adverse effects on normal tissues.

Clinical investigations in Europe and Asia have sparked interest in the use of heavier ions for cancer therapy, with the rationale that the higher RBE of heavy particles may be particularly valuable for overcoming the resistance of such tumors to photon irradiation^5^,^52^. The physical properties of protons have generally driven the worldwide expansion of proton therapy centers, but the inherent biologic differences between photon and proton beams have not been capitalized on to date. The LET values for the scanned proton beams in the current study are moderate in comparison to those for heavier ions. Interest in carbon ion therapy has increased because carbon ions have higher LET and higher biologic effectiveness, which could be useful for radiation-resistant tumors. However, the biologic uncertainties associated with heavy particles may be even greater than those of protons. Moreover, both the physical and biologic characteristics of protons and carbon ions may not make them the best particles for clinical use. Rather, intermediate particles may hold the greatest potential. Expanding the techniques developed here for use with other ion sources (helium, carbon, oxygen) would allow construction of a data matrix describing the interrelation of dose, LET, and biologic effect. In conjunction with computer modeling of physical properties, having such data could allow predictions of the biologic effect of other particles, which in turn could allow the most favorable particles to be identified before the costly construction of a therapy facility.

## Conclusion

Currently, in proton treatment planning, variable biological effectiveness is not formally accounted for; only physical properties are considered. Newer delivery technologies, such as spot scanning, allow the delivery of individually heterogeneous treatment fields by using techniques such as IMPT. In principle, optimization of IMPT could incorporate variable biological effectiveness to produce dose distributions in which protons with high biological effectiveness preferentially deposit dose in the tumor and the ones that pass through normal tissues are preferentially of low biological effectiveness. However, the substantial uncertainties associated with existing RBE data may preclude the development of accurate biologic models for use in such applications. By incorporating data generated using systems such as that described here, development of more accurate models and optimization of IMPT based on RBE may be feasible. In theory, this could enhance the therapeutic potential of particle therapy for numerous types of cancer.

## Methods

### Designing an irradiation device with Monte Carlo simulation

We used a calibrated and experimentally validated MC system based on the Geant4 toolkit to design the experimental device^53^,^54^. Three versions of Geant4 (9.5.p02, 10.0 and 10.1), with the pre-packaged FTFP_BERT (version 1.3 and 2.0) physics list, were tested and no differences were found for therapeutic proton simulations. The characteristics of proton beams entering the nozzle (e.g., energy, angular, and spatial spread) were fine-tuned so that the computed depth dose and beamlet profiles matched the corresponding measured data^55^. The thickness of each step of the irradiation jig (Fig. 1f) was selected from a 79.7-MeV spot-scanning beam depth-dose and depth-LET distribution curves in a Lucite phantom. The device was fabricated with a high-accuracy (±3 µm) milling machine. The original template was a cuboid block (21×19×11 cm^3^) of Lucite.

### Monte Carlo Dose and LET calculations in biological samples

A 5-µm cell layer in each well in the 96-well plate was considered to be the target for dose and LET calculations. Biologic effect is commonly assumed to be a function of dose-averaged LET (LET_d_), which was the case in this report as well. The number of primary source protons was set to 1.1x10^9^ to ensure that the statistical uncertainty in the calculated dose and LET values in the wells was ±<1%. LET was calculated on a step-by-step basis in the particle tracking process. The energy deposition *ε* over each proton step *l* within the cell layer was scored. Because of the stochastic nature of energy deposition by ionizing radiations, *ε*/*l* was treated as a random variable form of LET. The probability distribution of *ε*/*l* was scored during the simulation to evaluate the statistical uncertainty of LET calculation. In calculating LET_d_, *ε* was treated as the dose weighting factor of each *ε*/*l* of protons for each cell layer. A dynamic MC technique was used to simulate magnetic steering of the proton beamlet^56^. All MC simulations were carried out on our institutional high-performance computing cluster and the Lonestar cluster at the Texas Advanced Computational Center.

### Comparison of energy spectra for matching scattered and scanned proton beams

To illustrate the importance of using the scanning beam in contrast with the previous practice of using passively scattered beams, we selected a passively scattered beam of 120 MeV, which was broadened laterally with scatterers to form an 18 × 18 cm^2^ field and modulated longitudinally by a range modulation wheel to form an SOBP 3 cm wide. Finally, the beam was passed through the range shifter to achieve a range equal to that of the 79.7-MeV spot-scanning beam that we used for the biology experiments. Three points at depths of 2.15 cm, 4.0 cm, and 4.75 cm (positions A, B and C in Fig. 1a–c and Supplementary Fig. 1) along the beam path were selected to calculate and compare the proton kinetic energy spectra.

### Hitachi proton therapy system

Proton irradiation was done with the scanning beam gantry of the synchrotron and the Hitachi ProBeat delivery system (Hitachi, Ltd., Tokyo, Japan) at the Proton Therapy Center in Houston^57^. This delivery system can provide 94 discrete energies ranging from 72.5 MeV to 221.8 MeV^55^,^58^. It uses a step-and-shoot scanning technique in which the beamlet stops at a specified point and delivers the specified number of monitor units and then moves to the next position.

### Beam characteristics and scan patterns

The above-mentioned monoenergetic scanning beam of 79.7 MeV (range 4.8 cm in water and 4.1 cm in Lucite), with a spot of size 3.3 cm full-width-at-half-maximum in air at isocenter, was used for proton irradiation^55^. A 20 × 20 cm^2^ area was scanned uniformly. It had a 12 × 13 cm^2^ uniform high-dose region to ensure the wells in the plate periphery were sufficiently far from the penumbra to be affected by the lateral fall-off at the field edges (Fig. 2a&b). The spacing between spots was set to 1 cm in the isocenter plane. The spot intensity can be set to between 0.005 and 0.04 monitor units; we chose the maximum value for this study.

We used the rotational gantry with beam incident upon the bottom of the plate from below to minimize uncertainties arising from variability in the thickness of the fluid layer above the cells, setup, and scattering from well walls. Different incident dose levels were achieved by using multiple repaintings of the target plates with the scan patterns. The incident dose per repainting is determined through a calibration process described below. The relative dose levels per column in the 96-well plate were always maintained (Fig. 1e) to be the same for all irradiation experiments.

### System calibration and verification

We calibrated the system by using a calibrated plane-parallel ion chamber irradiated under reference conditions that excluded the jig. The dose at the calibrated chamber position under identical conditions was also calculated by MC simulations. The doses calculated in wells by MC simulations were normalized to the dose at the reference point. The calibration factor determined in this way led to the delivery of 2.6 cGy ± 0.1% per painting (requiring 17.64 monitor units) to the cell layer in column 1 in the presence of the jig. Each time before a set of cell irradiation experiments were done, quality assurance was done to ensure that the specified dose levels would be delivered.

### Positioning of the experimental devices

The geometric setting of the devices and samples was identical for all experiments. The jig, with the three films on top of it, is inserted into a holder, which is placed in the last downstream snout slot. The snout end was set to the same value, 9.1 ± 0.1 cm, for each irradiation, such that the top of the jig, where the biologic samples sit, was positioned at the isocenter plane (verified by laser cross markers from two orthogonal directions).

### Sensitivity analysis of experimental setup uncertainties

The Lucite jig, three EBT3 films, and well plate bottom constitute the energy-attenuating components for protons before the cell layer. Hence, the MC-calculated accuracy of the delivered dose and LET values depends on the thickness, chemical composition, and density of these materials, especially for wells located near the end of the beam’s range.

Notably, given the geometry we use and the uniformity of the field of irradiation, the accuracy of the dose delivered to the samples is insensitive to small changes in position longitudinally or laterally relative to the beam. It is almost entirely a function of precision of the thicknesses of the jig steps, well plate, films, and the accuracy of the knowledge the material densities and compositions. Because we used the same jig and films for all experiments, they do not contribute to random uncertainties, but they may contribute to systematic uncertainties. However, since the jig was fabricated with a high-accuracy milling machine and the vendor-quoted uncertainty in film thickness is very small (Supplementary Table 2), the overall systematic uncertainty in dose was estimated to be negligible. However, the scattering properties of the jig were verified by measuring the dose and range of the transmitted beam using with films and ion chamber and comparing the results with MC simulations.

Because large numbers of plates were used, manufacturing variability in the thickness of the well bottom and its composition could contribute non-negligible uncertainty to dose in the high-gradient distal fall-off region. The chamber and MC simulations each contributed less than ±1% to uncertainty.

Parameters used for estimating uncertainties are given in Supplementary Table 2. The nominal density of the jig material and 96-well plate material was set to 1.19 and 1.09 g/cm^3^ 59,60. The estimated uncertainties, dominated by the random component, are given in Supplementary Table 3. The highest uncertainties correspond to the high-dose gradient at the distal edge.

The MC-calculated dose and LET using the nominal value were treated as the mean values (Fig. 1e). The average deviation from the nominal with lower and upper ranges of setups was treated as the uncertainty in the nominal value to yield the uncertainty in dose and LET values (error bars in Fig. 1e, Supplementary Table 3). The effect of uncertainties on protons energy spectra in three of the columns of the 96-well plate are depicted in Supplementary Fig. 2.

### Biologic sample preparation, irradiation, and processing

H460 and H1437 NSCLC cells were cultured in RPMI 1640 medium with 10% fetal bovine serum and 1% penicillin-streptomycin-L-glutamine at 37°C and 5% CO_2_. Cells were counted using an automated cell counter and seeded at concentrations ranging from 100–2000 cells per well for the standard 6-well clonogenic assay and at 100 cells per well when using the 96-well format. Plating reproducibility was ensured by using a BioTek MultiFlo FX Microplate Dispenser for automated and rapid cell plating. Cells were allowed to attach and stabilize in culture for 8–10 hours before irradiation. Plates were brought into the treatment room one at a time for irradiation and immediately returned to culture after exposure. Control plates were handled identically to treatment plates but not irradiated. Two plates per dose level were irradiated, resulting in 16 replicates per LET-dose combination. After colonies formed (at 5 days for the H460 cells and 7 days for H1437 cells), cells were fixed and stained with 0.5% crystal violet in methanol. High-content automated laser confocal analysis with an IN Cell Analyzer 6000 was used to identify viable colonies containing ≥50 cells. Briefly, using a 4 × objective, four overlapping fields per well were obtained and the GE Developer v1.9 software used to create a composite. Colonies and cells were identified with masks generated from object filters. Cells were linked to colonies, and only colonies containing 50 or more cells were scored. The excitation wavelength was 640 nm (red) and the emission wavelength was 706 nm (Cy5). The 4 × lens has a 0.20 numerical aperture. The IN Cell uses a 5.5-Mp sCMOS camera (2560 × 2160 pixels) with a 6.5-µm pixel size. We defined the limit of detection for a clonogenic screen assay as 1 colony per well or an SF of 1/(cells plated*plating efficiency). Dose levels where the aggregate SF was lower than the limit were omitted from analysis. SFs were analyzed by normalizing the number of counted colonies at a given dose by the plating efficiency and by fitting the obtained data to a linear-quadratic model using weighted non-linear regression.

Dose-LET-dependent γH2AX foci formation after proton irradiation was examined by plating cells into a glass-bottom microplate, irradiating them using the high-throughput system, and returning to culture. Two hours after irradiation, cells were fixed with 4% paraformaldehyde in phosphate buffered saline (PBS). The fixative was removed and cells were washed in PBS 3 times before permeabilization with 0.5% Triton X-100/PBS. Permeabilized cells were then blocked with a 5% goat serum/0.3% Triton X-100/PBS solution. For primary labeling, the cells were incubated with a murine γH2AX antibody (1:1000, clone JBW301, EMD Millipore) in 1% bovine serum albumin (BSA)/0.1% Triton X-100/PBS. Cells were then washed 3 times with 0.1% Triton X-100/PBS and incubated with AlexaFluor 488-labeled goat anti-mouse antibody in 1% BSA/0.1% Triton X-100/PBS (1:1000, Life Technologies). The cells were washed again with 0.1% Triton X-100/PBS before mounting medium was added with the fluorescent dye 4',6-diamidino-2-phenylindole (DAPI). Plates were imaged on an Olympus IX81 microscope using a 40x water immersion objective. The average number of γH2AX foci per nucleus was determined by using CellProfiler (Broad Institute) to identify DAPI-labeled nuclei as image masks and quantify the number of associated γH2AX foci. Images having the same average and median number of foci per nucleus matching the respective condition’s overall pooled value were selected as the representative images.

### Statistical analyses

Statistical analyses were done using GraphPad Prism 6.0. SF data are shown on a semilog scale as mean ± standard error of the mean. Data were fit using a weighted (1/Y)-nonlinear regression to the linear-quadratic model. The extra sum-of-squares *F* test was used to compare clonogenic survival curves as a function of LET. The γH2AX foci data are shown as mean ±95% confidence interval. The average numbers of γH2AX foci for each condition were tested for statistical significance by the Mann-Whitney unpaired *t* test. RBE standard deviations were calculated by propagating the standard error of the α and β fits.

## Additional Information

**How to cite this article**: Guan, F. *et al*.Spatial mapping of the biologic effectiveness of scanned particle beams: towards biologically optimized particle therapy. *Sci. Rep.* **5**, 9850; doi: 10.1038/srep09850 (2015).

## Supplementary Material

Supplementary Information Supplementary Information

## Figures and Tables

**Table 1:** α and β fit parameters and RBE values at 10% surviving fraction.

Cell line:		H460			H1437	
LET [keV/μm]	α	β	RBE	α	β	RBE
0.9	0.268	0.097	1.03	0.077	0.028	0.89
1.2	0.226	0.112	1.04	0.136	0.020	0.88
1.6	0.151	0.134	1.05	0.067	0.027	0.85
1.8	0.150	0.134	1.05	0.059	0.038	0.98
1.9	0.166	0.134	1.06	0.094	0.031	0.96
2.3	0.137	0.146	1.07	0.096	0.032	0.97
3.0	0.206	0.125	1.07	0.111	0.033	1.02
5.1	0.117	0.159	1.10	0.034	0.052	1.10
10.8	0.318	0.154	1.28	0.119	0.054	1.26
15.2	0.446	0.341	1.87	0.180	0.095	1.70
17.7	0.596	0.662	2.58	0.328	0.149	2.33
19.0	0.883	0.956	3.28	0.360	0.272	2.98
Photons(^137^Cs)	0.290	0.083	1.00	0.050	0.041	1.00

## References

[b1] Schulz-Ertner D. & Tsujii H. Particle radiation therapy using proton and heavier ion beams. J Clin Oncol 25, 953–964, 10.1200/JCO.2006.09.7816 (2007). 17350944

[b2] Allen A. M. *et al.* An evidence based review of proton beam therapy: the report of ASTRO's emerging technology committee. Radiother Oncol 103, 8–11, 10.1016/j.radonc.2012.02.001 (2012). 22405807

[b3] Wedenberg M. & Toma-Dasu I. Disregarding RBE variation in treatment plan comparison may lead to bias in favor of proton plans. Med Phys 41, 091706, 10.1118/1.4892930 (2014). 25186381

[b4] Halperin E. C. Particle therapy and treatment of cancer. Lancet Oncol 7, 676–685, 10.1016/s1470-2045(06)70795-1 (2006). 16887485

[b5] Combs S. E. *et al.* Comparison of carbon ion radiotherapy to photon radiation alone or in combination with temozolomide in patients with high-grade gliomas: explorative hypothesis-generating retrospective analysis. Radiother Oncol 108, 132–135, 10.1016/j.radonc.2013.06.026 (2013). 23932193

[b6] Schardt D., Elsaesser T. & Schulz-Ertner D. Heavy-ion tumor therapy: Physical and radiobiological benefits. Reviews of Modern Physics 82 10.1103/RevModPhys.82.383 (2010).

[b7] Burigo L., Pshenichnov I., Mishustin I. & Bleicher M. Comparative study of RBE and cell survival fractions for ^1^H, ^4^He, ^12^C and ^16^O beams using Geant4 and Microdosimetric Kinetic model. arXiv preprint arXiv*;* 1403.7929 (2014). 10.1088/0031-9155/60/8/3313 25825827

[b8] Paganetti H. *et al.* Relative biological effectiveness (RBE) values for proton beam therapy. Int J Radiat Oncol Biol Phys. 53, 407–421. (2002). 12023146 10.1016/s0360-3016(02)02754-2

[b9] Paganetti H. Relative biological effectiveness (RBE) values for proton beam therapy. Variations as a function of biological endpoint, dose, and linear energy transfer. Phys Med Biol 59, R419–472, 10.1088/0031-9155/59/22/R419 (2014). 25361443

[b10] Britten R. A. *et al.* Variations in the RBE for Cell Killing Along the Depth-Dose Profile of a Modulated Proton Therapy Beam. Radiat Res 13, 13 (2012). 10.1667/RR2737.1 23148508

[b11] Calugaru V. *et al.* Radiobiological characterization of two therapeutic proton beams with different initial energy spectra used at the Institut Curie Proton Therapy Center in Orsay. Int J Radiat Oncol Biol Phys 81, 1136–1143, 10.1016/j.ijrobp.2010.09.003 (2011). 21075549

[b12] Chaudhary P. *et al.* Relative Biological Effectiveness Variation Along Monoenergetic and Modulated Bragg Peaks of a 62-MeV Therapeutic Proton Beam: A Preclinical Assessment. Int J Radiat Oncol Biol Phys, 10.1016/j.ijrobp.2014.05.010 (2014). 24986743

[b13] Britten R. A. *et al.* Variations in the RBE for cell killing along the depth-dose profile of a modulated proton therapy beam. Radiat Res 179, 21–28, 10.1667/RR2737.1 (2013). 23148508

[b14] Lin S. H. *et al.* A high content clonogenic survival drug screen identifies mek inhibitors as potent radiation sensitizers for KRAS mutant non-small-cell lung cancer. J Thorac Oncol 9, 965–973, 10.1097/JTO.0000000000000199 (2014). 24922006 PMC4110054

[b15] Lee S. J., Lee H. J. & Moon D. H. Quantitative analysis of thymidine kinase 1 and 5'(3')-deoxyribonucleotidase mRNA expression: the role of fluorothymidine uptake. Anticancer Res 31, 2135–2139 (2011). 21737633

[b16] Carmichael J., DeGraff W. G., Gazdar A. F., Minna J. D. & Mitchell J. B. Evaluation of a tetrazolium-based semiautomated colorimetric assay: assessment of chemosensitivity testing. Cancer Res 47, 936–942 (1987). 3802100

[b17] Lai S. L., Perng R. P. & Hwang J. p53 gene status modulates the chemosensitivity of non-small cell lung cancer cells. J Biomed Sci 7, 64–70, 25431 (2000). 10644891 10.1007/BF02255920

[b18] Puck T. T. & Marcus P. I. Action of x-rays on mammalian cells. J Exp Med 103, 653–666 (1956). 13319584 10.1084/jem.103.5.653 PMC2136626

[b19] Franken N. A., Rodermond H. M., Stap J., Haveman J. & van Bree C. Clonogenic assay of cells in vitro. Nat Protoc 1, 2315-2319, 10.1038/nprot.2006.339 (2006). 17406473

[b20] Chamberlain A. C. & Turner F. M. Errors and Variations in White-Cell Counts. Biometrics 8, 55–65, 10.2307/3001526 (1952).

[b21] Willén E. A simplified method of phytoplankton counting. British Phycological Journal 11, 265–278, 10.1080/00071617600650551 (1976).

[b22] Gerweck L. E., Dullea R., Zaidi S. T., Budach W. & Hartford A. Influence of experimental factors on intrinsic radiosensitivity assays at low doses of radiation: cell multiplicity. Radiat Res 138, 361–366 (1994). 8184010

[b23] Voger E. A. & Bussian R. W. Short-term cell-attachment rates: a surface-sensitive test of cell-substrate compatibility. J Biomed Mater Res 21, 1197–1211, 10.1002/jbm.820211004 (1987). 3693384

[b24] Twentyman P. R. Timing of assays: an important consideration in the determination of clonogenic cell survival both in vitro and in vivo. Int J Radiat Oncol Biol Phys 5, 1213–1220 (1979). 528265 10.1016/0360-3016(79)90641-2

[b25] Wu R. C. & Schonthal A. H. Activation of p53-p21waf1 pathway in response to disruption of cell-matrix interactions. J Biol Chem 272, 29091–29098 (1997). 9360984 10.1074/jbc.272.46.29091

[b26] Lewis J. M., Truong T. N. & Schwartz M. A. Integrins regulate the apoptotic response to DNA damage through modulation of p53. Proc Natl Acad Sci U S A 99, 3627–3632, 10.1073/pnas.062698499 (2002). 11904424 PMC122574

[b27] Kang M. A., So E. Y. & Ouchi T. Deregulation of DNA damage response pathway by intercellular contact. J Biol Chem 287, 16246–16255, 10.1074/jbc.M111.337212 (2012). 22431734 PMC3351312

[b28] Kapiszewska M., Reddy N. M. & Lange C. S. Trypsin-induced changes in cell shape and chromatin structure result in radiosensitization of monolayer Chinese hamster V79 cells. Int J Radiat Biol 60, 635–646 (1991). 1680144 10.1080/09553009114552461

[b29] Huang H. L. *et al.* Trypsin-induced proteome alteration during cell subculture in mammalian cells. J Biomed Sci 17, 36, 10.1186/1423-0127-17-36 (2010). 20459778 PMC2873939

[b30] Ogata T. *et al.* Particle irradiation suppresses metastatic potential of cancer cells. Cancer Res 65, 113–120 (2005). 15665286

[b31] Akino Y. *et al.* Carbon-ion beam irradiation effectively suppresses migration and invasion of human non-small-cell lung cancer cells. Int J Radiat Oncol Biol Phys 75, 475–481, 10.1016/j.ijrobp.2008.12.090 (2009). 19735871

[b32] Suetens A. *et al.* Dose- and time-dependent gene expression alterations in prostate and colon cancer cells after in vitro exposure to carbon ion and X-irradiation. J Radiat Res, 10.1093/jrr/rru070 (2014). PMC4572596 25190155

[b33] van Bree C. *et al.* G0 cell cycle arrest alone is insufficient for enabling the repair of ionizing radiation-induced potentially lethal damage. Radiat Res 170, 184–191, 10.1667/RR0845.1 (2008). 18666809

[b34] Barendsen G. W., Van Bree C. & Franken N. A. Importance of cell proliferative state and potentially lethal damage repair on radiation effectiveness: implications for combined tumor treatments (review). Int J Oncol 19, 247–256 (2001). 11445835 10.3892/ijo.19.2.247

[b35] Little J. B. Factors influencing the repair of potentially lethal radiation damage in growth-inhibited human cells. Radiat Res 56, 320–333 (1973). 4749594

[b36] Baskar R. Emerging role of radiation induced bystander effects: Cell communications and carcinogenesis. Genome Integr 1, 13, 10.1186/2041-9414-1-13 (2010). 20831828 PMC2949714

[b37] Belli M. *et al.* Inactivation of human normal and tumour cells irradiated with low energy protons. Int J Radiat Biol 76, 831–839 (2000). 10902738 10.1080/09553000050028995

[b38] Belli M. *et al.* RBE-LET relationships for cell inactivation and mutation induced by low energy protons in V79 cells: further results at the LNL facility. Int J Radiat Biol 74, 501–509 (1998). 9798961 10.1080/095530098141375

[b39] ICRU Report 86, Quantification and Reporting of Low-Dose and other Heterogeneous Exposures. . Journal of the ICRU 11, 51–60 (2011). 10.1093/jicru/ndr028 24174422

[b40] Petrovic I. *et al.* Response of a radioresistant human melanoma cell line along the proton spread-out Bragg peak. Int J Radiat Biol 86, 742–751, 10.3109/09553002.2010.481322 (2010). 20597839

[b41] Freedman V. H. & Shin S. I. Cellular tumorigenicity in nude mice: correlation with cell growth in semi-solid medium. Cell 3, 355–359 (1974). 4442124 10.1016/0092-8674(74)90050-6

[b42] Fiebig H. H., Maier A. & Burger A. M. Clonogenic assay with established human tumour xenografts: correlation of in vitro to in vivo activity as a basis for anticancer drug discovery. Eur J Cancer 40, 802–820, 10.1016/j.ejca.2004.01.009 (2004). 15120036

[b43] Scholz C. C., Berger D. P., Winterhalter B. R., Henss H. & Fiebig H. H. Correlation of drug response in patients and in the clonogenic assay with solid human tumour xenografts. Eur J Cancer 26, 901–905 (1990). 2145936 10.1016/0277-5379(90)90196-z

[b44] Fertil B. & Malaise E. P. Intrinsic radiosensitivity of human cell lines is correlated with radioresponsiveness of human tumors: analysis of 101 published survival curves. Int J Radiat Oncol Biol Phys 11, 1699–1707 (1985). 4030437 10.1016/0360-3016(85)90223-8

[b45] Michalowski A. A critical appraisal of clonogenic survival assays in the evaluation of radiation damage to normal tissues. Radiother Oncol 1, 241–246 (1984). 6505260 10.1016/s0167-8140(84)80006-7

[b46] Hall E. J. & Giaccia A. J. Radiobiology for the radiologist. 6th edn, (Lippincott Williams & Wilkins, 2006).

[b47] Wilkens J. J. & Oelfke U. Optimization of radiobiological effects in intensity modulated proton therapy. Med Phys 32, 455–465 (2005). 15789592 10.1118/1.1851925

[b48] Tibbitt M. W. & Anseth K. S. Hydrogels as extracellular matrix mimics for 3D cell culture. Biotechnol Bioeng 103, 655–663, 10.1002/bit.22361 (2009). 19472329 PMC2997742

[b49] Griffith L. G. & Swartz M. A. Capturing complex 3D tissue physiology in vitro. Nat Rev Mol Cell Biol 7, 211–224, 10.1038/nrm1858 (2006). 16496023

[b50] Howes A. L., Richardson R. D., Finlay D. & Vuori K. 3-Dimensional culture systems for anti-cancer compound profiling and high-throughput screening reveal increases in EGFR inhibitor-mediated cytotoxicity compared to monolayer culture systems. PLoS One 9, e108283, 10.1371/journal.pone.0108283 (2014). 25247711 PMC4172726

[b51] Chambers K. F., Mosaad E. M., Russell P. J., Clements J. A. & Doran M. R. 3D cultures of prostate cancer cells cultured in a novel high-throughput culture platform are more resistant to chemotherapeutics compared to cells cultured in monolayer. PLoS One 9, e111029, 10.1371/journal.pone.0111029 (2014). 25380249 PMC4224379

[b52] Mizoe J. E. *et al.* Phase I/II clinical trial of carbon ion radiotherapy for malignant gliomas: combined X-ray radiotherapy, chemotherapy, and carbon ion radiotherapy. Int J Radiat Oncol Biol Phys 69, 390–396, 10.1016/j.ijrobp.2007.03.003 (2007). 17459607

[b53] Agostinelli S. *et al.* Geant4—a simulation toolkit. Nuclear Instruments and Methods in Physics Research Section A 506, 250–303 (2003).

[b54] Allison J. *et al.* Geant4 developments and applications. IEEE TRANSACTIONS ON NUCLEAR SCIENCE 53, 270-278 (2006).

[b55] Zhu X. R. *et al.* Commissioning dose computation models for spot scanning proton beams in water for a commercially available treatment planning system. Med Phys 40, 041723, 10.1118/1.4798229 (2013). 23556893 PMC3631269

[b56] Paganetti H. Four-dimensional Monte Carlo simulation of time-dependent geometries. Phys Med Biol 49, N75-81 (2004). 15104330 10.1088/0031-9155/49/6/n03

[b57] Smith A. *et al.* The M. D. Anderson proton therapy system. Med Phys 36, 4068–4083 (2009). 19810479 10.1118/1.3187229

[b58] Gillin M. T. *et al.* Commissioning of the discrete spot scanning proton beam delivery system at the University of Texas M.D. Anderson Cancer Center, Proton Therapy Center, Houston. Med Phys 37, 154–163 (2010). 20175477 10.1118/1.3259742 PMC11078095

[b59] Haynes W. M. CRC Handbook of Chemistry and Physics, 95th Edition. (CRC Press., 2014).

[b60] Berger M. J., Coursey J. S., Zucker M. A. & Chang J. ESTAR, PSTAR, and ASTAR: Computer Programs for Calculating Stopping-Power and Range Tables for Electrons, Protons, and Helium Ions (version 1.2.3). [Online] Available: http://physics.nist.gov/Star [2014, July 23]National Institute of Standards and Technology, Gaithersburg, MD. (2005).

