# Supplementary material for: Spatial mapping of the biologic effectiveness of scanned particle beams: towards biologically optimized particle therapy
Source: Sci Rep. 2015 May 18;5:9850. doi: 10.1038/srep09850 (PMC4650781; doi:10.1038/srep09850)
Supplement: Supplementary Information [file srep09850-s1.pdf]

Supplementary Materials

Spatial mapping of the biologic effectiveness of scanned particle beams: towards biologically optimized particle therapy

Fada Guan[1,*], Lawrence Bronk[2,*], Uwe Titt[1], Steven H. Lin[2,3], Dragan Mirkovic[1], Matthew D. Kerr[1], X. Ronald Zhu[1], Jeffrey Dinh[2], Mary Sobieski[4], Clifford Stephan[4], Christopher R. Peeler[1], Reza Taleei[1], Radhe Mohan[1] and David R. Grosshans[2,3]

Departments of [1]Radiation Physics, [2]Experimental Radiation Oncology, and [3]Radiation Oncology, The University of Texas MD Anderson Cancer Center, Houston, Texas, USA; and [4]Center for Translational Cancer Research, Texas A&M Health Science Center, Institute of Biosciences and Technology, Houston, Texas, USA.

[*] These authors contributed equally to this work.

Correspondence should be addressed to D.R.G. (dgrossha@mdanderson.org) or R.M. (rmohan@mdanderson.org)

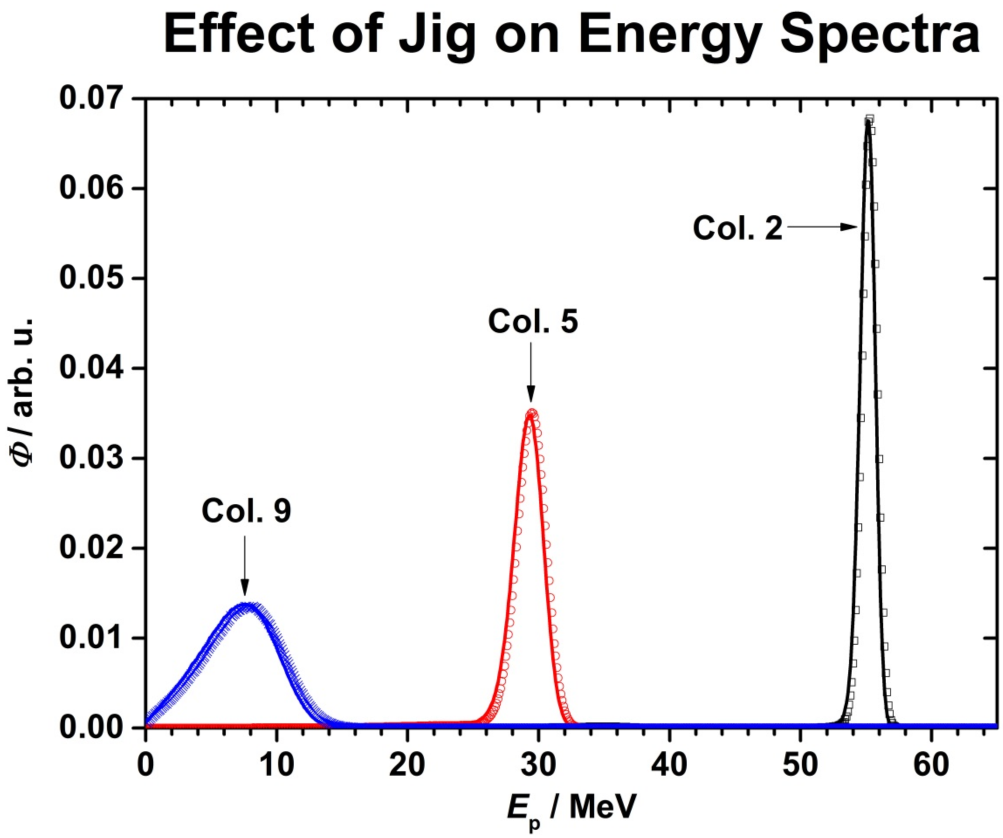

Supplementary Figure 1. Comparison of energy spectra of 79.7-MeV scanned proton beam with the jig and at equivalent depths without the jig shows that the spectra were not perceptibly altered by scattering from the steps of the jig. Symbols depict energy spectra at the sample positions in the wells for three of the columns (2, 5 and 9) with the jig (plus 3 EBT3 films) in place. The solid lines represent the spectra in the absence of the jig at the equivalent depths in a homogeneous phantom. The differences are negligible, with mean energy shifts with and without the jig being +0.2 MeV, +0.2 MeV, and +0.4 MeV in the three columns.

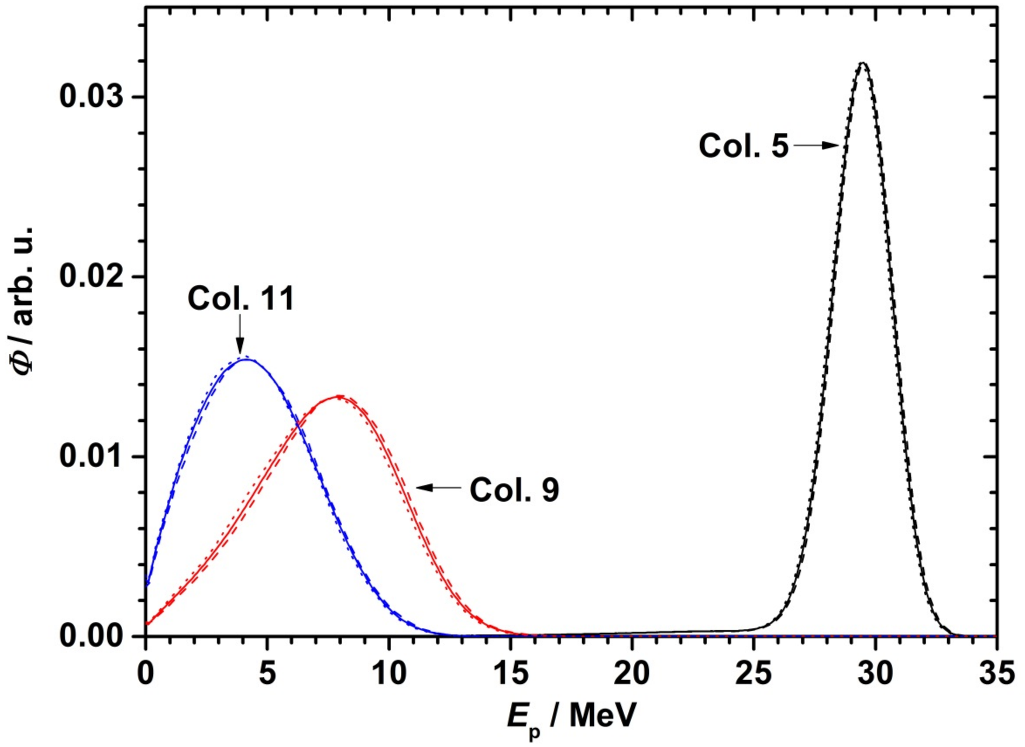

Supplementary Figure 2. The effect of uncertainties estimated via sensitivity analysis on energy spectra. Solid lines represent the energy spectra obtained using the nominal setup of the experimental devices; dashed lines represent the energy spectra obtained with the lowest estimate of uncertainty; and dotted lines represent the energy spectra obtained with the highest estimate. Although the profiles of the energy spectra are maintained in the face of uncertainties, small mean energy shifts were observed as expected.

Supplementary Table 1. 95% confidence intervals for α and β fit parameters.

**Cell line:**	**H460**		**H1437**	
**LET [keV/μm]**	**α**	**β**	**α**	**β**
0.9	0.239, 0.297	0.0829, 0.112	0.0, 0.173	0.0, 0.0655
1.2	0.199, 0.252	0.0996, 0.124	0.0590, 0.212	0.0, 0.0446
1.6	0.126, 0.176	0.123, 0.146	0.0253, 0.108	0.0152, 0.0382
1.8	0.124, 0.175	0.122, 0.147	0.0198, 0.0983	0.0272, 0.0481
1.9	0.139, 0.193	0.121, 0.148	0.0562, 0.131	0.0213, 0.0403
2.3	0.106, 0.169	0.129, 0.162	0.0617, 0.131	0.0235, 0.0398
3.0	0.172, 0.240	0.110, 0.141	0.0683, 0.153	0.0229, 0.0441
5.1	0.0796, 0.155	0.144, 0.175	0.0, 0.0840	0.0400, 0.0646
10.8	0.273, 0.363	0.136, 0.171	0.0537, 0.184	0.0385, 0.0705
15.2	0.380, 0.513	0.302, 0.379	0.0959, 0.265	0.0677, 0.121
17.7	0.512, 0.680	0.595, 0.729	0.211, 0.445	0.0976, 0.200
19.0	0.776, 0.989	0.842, 1.07	0.220, 0.500	0.186, 0.357
Photons(^{137}Cs)	0.259, 0.322	0.0757, 0.0912	0.0273, 0.0723	0.0364, 0.0450

Supplementary Table 2. Parameters for uncertainty analysis.

	**Lower range**	**Nominal values**	**Upper range**
Jig step thickness (μm)	-5	designed values	+5
EBT3 film thickness (μm)	-1	268	+1
Well bottom thickness (μm)	-15	1240	+15
Well plate material polystyrene density (g/cm^3)	-0.01	1.09	+0.01

Supplementary Table 3. Average relative uncertainty bounds (%) for dose and LET.

Column #	Dose	LET
1	±0.3%	±0.1%
2	±0.4%	±0.9%
3	±0.6%	±0.3%
4	±0.3%	±0.3%
5	±0.2%	±0.0%
6	±0.3%	±0.4%
7	±0.6%	±0.5%
8	±1.2%	±1.2%
9	±0.0%	±2.3%
10	±3.5%	±1.3%
11	±6.7%	±1.1%
12	±8.6%	±2.4%
